# Computational Optimization of the 3D Least-Squares Matching Algorithm by Direct Calculation of Normal Equations

**DOI:** 10.3390/tomography8020063

**Published:** 2022-03-14

**Authors:** Frank Liebold, Hans-Gerd Maas

**Affiliations:** Institute of Photogrammetry and Remote Sensing, Technische Universität Dresden, 01062 Dresden, Germany; hans-gerd.maas@tu-dresden.de

**Keywords:** 3D least-squares matching, cuboid tracking, 3D displacement field, voxel data, microtomography data

## Abstract

3D least-squares matching is an algorithm that allows to measure subvoxel-precise displacements between two data sets of computed tomography voxel data. The determination of precise displacement vector fields is an important tool for deformation analyses in in-situ X-ray micro-tomography time series. The goal of the work presented in this publication is the development and validation of an optimized algorithm for 3D least-squares matching saving computation time and memory. 3D least-squares matching is a gradient-based method to determine geometric (and optionally also radiometric) transformation parameters between consecutive cuboids in voxel data. These parameters are obtained by an iterative Gauss-Markov process. Herein, the most crucial point concerning computation time is the calculation of the normal equations using matrix multiplications. In the paper at hand, a direct normal equation computation approach is proposed, minimizing the number of computation steps. A theoretical comparison shows, that the number of multiplications is reduced by 28% and the number of additions by 17%. In a practical test, the computation time of the 3D least-squares matching algorithm was proven to be reduced by 27%.

## 1. Introduction

**Figure 1:**
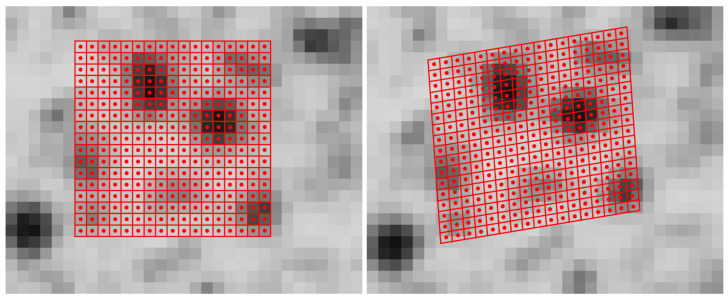
Least-squares matching example: (**a**) reference image with the red colored patch; (**b**) second (warped) image with the red colored transformed patch.

Volume data analysis in a voxel space representation is a logical extension of 2D image data processing. In the field of Photogrammetry and Computer Vision, image correlation techniques are often used for fine measurement of corresponding points in image sequences or stereo image pairs. Early contributions applied cross-correlation to image data [1]. In the early 80’s, gradient based algorithms were developed using an iterative least squares method [2] that also offers subpixel precision. Lucas and Kanade [2] only introduced two translation parameters, whereas Ackermann [3] and Grün [4] extended the model toward an affine and radiometric transformation, which also considers rotation, scaling and shear to obtain a better adaption for perspective distortions. As an example for the least-squares matching (LSM) algorithm, an image section of a textured concrete surface with two views is illustrated in Figure 1. Figure 1a shows a section of the reference image with the patch (subimage) consisting of the pixels included in the computation. The pixels are illustrated as red squares with red circles in the centers. In Figure 1b, an image section of the second view as well as the transformed patch (red squares with red circles) are depicted. In this example, the transformation includes translation, rotation, scale and shear and the corresponding parameters are obtained by the LSM algorithm. The most important parameters are the shifts, which typically show an accuracy of 0.02 to 0.05 pixels in this kind of application.

It is also possible to use more complex geometric models for the perspective transformation [5]. Grün and Baltsavias [6] introduced geometrically constrained multiphoto matching, where the solution can inherently be forced to the epipolar line. Other contributions combined digital image matching and object surface reconstruction. In addition to a geometric 3D model of the surface, they used a radiometric model for the surface and the images. The unknowns of the surface densities, 3D points and image orientation parameters were computed in an iterative least-squares adjustment [7,8].

Maas et al. [9] transferred the idea from the 2D image matching to 3D voxel data (digital volume correlation DVC). They applied the subvoxel-precise cuboid tracking (3D least-squares matching) with 12 parameters of a 3D affine transformation to a sequence of multi-temporal voxel representations of mixing fluids. Bay et al. [10] extended the cross-correlation technique to 3D, combined it with a subvoxel-precise refinement and applied it to X-ray tomography data for strain analysis. Liebold et al. [11] also introduced a radiometric transformation to 3D cuboid matching.

In the last years, also free DVC code was provided. For instance, TomoWarp2 ensures the computation of 3D displacement fields using 3D cross-correlation [12]. They also implemented a further step to calculate subvoxel positions considering the maximum cross-correlation integer position and the 26 neighbor voxel around. The free software package SPAM is based on TomoWarp2 [13] and also provides a tool for the coarse registration that can consider a rotation between the reference and the deformed volume. In ALDVC [14], a Fast Fourier Transform (FFT) is applied for the estimation of initial shift values, followed by an algorithm including a 12-parameter affine transformation similar to 3D least-squares matching for computing subvoxel positions.

The least-squares matching algorithm contains an adjustment procedure where normal equations are computed. To speed up the process, Sutton et al. [15] showed a direct computation of the normal equations for 2D least-squares matching including affine parameters, and Jin et al. [16] also included radiometric parameters.

The publication at hand is based on the work of Maas et al. [9] and Liebold et al. [11]. It presents an optimized algorithm for the 3D LSM, likewise to what has been shown for 2D [15,16]. The central goal of the work is the acceleration of the 3D algorithm in order to save computation time and memory, without restrictions to the universality of the algorithm. The paper is structured as follows: In the next section, an overview of the computation of 3D least-squares matching is given including the optimization. In addition, a comparison to the standard method is shown. The publication closes with a conclusion.

## 2. 3D Least-Squares Matching

### 2.1. Mathematical Model

In the following, some formulae of Liebold et al. [11] are reused and extended. 3D least-squares matching is a gradient-based method and is applied to two voxel data sets. In the reference volume, a small cuboid is defined containing the voxel of the cuboid’s center (point to be matched) and its neighborhood. The dimensions $dim_{x}$, $dim_{y}$ and $dim_{z}$ are odd numbers, and a typical cuboid size is for example 13×13×13=2197 voxel. The aim is to find the corresponding cuboid in the second volume data set. The coordinates of the center of the cuboid in the reference state $x_{Ref,c},y_{Ref,c},z_{Ref,c}$ are integer values, whereas the coordinates of the voxel in the second state $x_{Def},y_{Def},z_{Def}$ are floating-point values.

**Figure 2:**
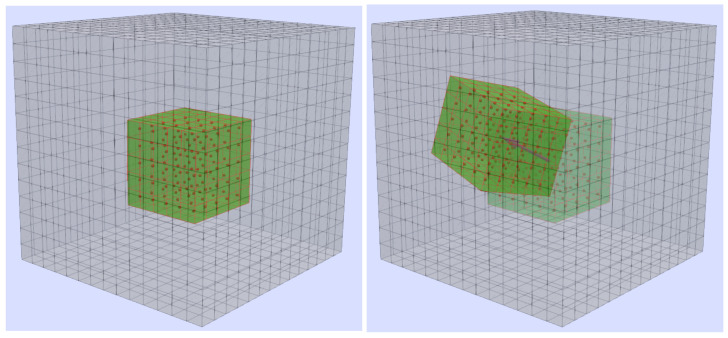
(**a**) Reference voxel grid with the subvolume including the voxel (green with red edges and red center points) that are used for the computation. (**b**) Second volume with the transformed subvolume. The shift vector is colored in magenta and the reference subvolume is faded.

Similar to Figure 1 for 2D, Figure 2 illustrates the 3D case with a voxel grid (gray). In the reference state, a subvolume consisting of e.g., 5×5×5 voxel (green cuboid with red edges and red center points, Figure 2a). In the second state, depicted in Figure 2b, the affine-transformed cuboid as well as the faded reference subvolume are shown. The shift vector that connects the center points of the subvolumes is colored in magenta. 3D LSM is used to determine the transformation parameters, including the three translations.

The range of the cuboid in the reference volume is:

(1) $$\begin{array}{cccc} \; & x_{Ref}\in \left[x_{Ref,c}-h_{x},x_{Ref,c}+h_{x}\right] & \wedge & x_{Ref}\in N\\ \; & y_{Ref}\in \left[y_{Ref,c}-h_{y},y_{Ref,c}+h_{y}\right] & \wedge & y_{Ref}\in N\\ \; & z_{Ref}\in \left[z_{Ref,c}-h_{z},z_{Ref,c}+h_{z}\right] & \wedge & z_{Ref}\in N \end{array}$$

where $h_{x}=\frac{{dim}_{x}-1}{2}$; $h_{y}=\frac{{dim}_{y}-1}{2}$; $h_{z}=\frac{{dim}_{z}-1}{2}$

Equation (2) shows the relationship between the gray values of the first (reference) and second (deformed) volume, also taking into account radiometric parameters ($r_{0},r_{1}$). This relationship is valid for each voxel of the cuboid.

(2) $$f\left(x_{Ref},y_{Ref},z_{Ref}\right)=r_{0}+r_{1}\cdot g\left(x_{Def},y_{Def},z_{Def}\right)$$

where

$x_{Ref},y_{Ref},z_{Ref}=\;coordinates (reference state)$
$x_{Def},y_{Def},z_{Def}=\;\mathrm{coordinates (deformed state)}$
$f(x_{Ref},y_{Ref},x_{Ref})=\;gray value (reference state)$
$g(x_{Def},y_{Def},z_{Def})=\;gray value (deformed state)$
$r_{0},r_{0}=\;brightness and contrast parameters$

Between the reference and the deformed state, a coordinate transformation is performed. Similar to [3] in 2D and [9] in 3D, an affine transformation is used as mathematical model containing displacements, rotations, scaling and shear, see Equation (3), where $a_{0},b_{0}$ and $c_{0}$ are the translation parameters.

(3) $$\begin{array}{ccc} x_{Def} & = & x_{Ref,c}+a_{0}+a_{1}\cdot \tilde{x}+a_{2}\cdot \tilde{y}+a_{3}\cdot \tilde{z}\\ y_{Def} & = & y_{Ref,c}+b_{0}+b_{1}\cdot \tilde{x}+b_{2}\cdot \tilde{y}+b_{3}\cdot \tilde{z}\\ z_{Def} & = & z_{Ref,c}+c_{0}+c_{1}\cdot \tilde{x}+c_{2}\cdot \tilde{y}+c_{3}\cdot \tilde{z} \end{array}$$

where

$a_{i},b_{i},c_{i}=\;parameters of the affine transformation$
$\tilde{x},\tilde{y},\tilde{z}=\;reduced reference coordinates$

The reduced reference coordinates are computed as follows:

(4) $$\begin{array}{ccc} \tilde{x} & = & x_{Ref}-x_{Ref,c}\\ \tilde{y} & = & y_{Ref}-y_{Ref,c}\\ \tilde{z} & = & z_{Ref}-z_{Ref,c} \end{array}$$

Thus, the vector of unknowns is:

(5) $$p={\left(\begin{array}{cccccccccccccc} a_{0}, & a_{1}, & a_{2}, & a_{3}, & b_{0}, & b_{1}, & b_{2}, & b_{3}, & c_{0}, & c_{1}, & c_{2}, & c_{3}, & r_{0}, & r_{1} \end{array}\right)}^{T}$$

The parameter vector p consists of 14 unknowns, and a cuboid with an exemplary size of 13×13×13=2197 voxel (observations) leads to an over-determination. Thus, the parameters of p are computed in a least-squares adjustment process. Therefore, residuals are added to Equation (2) that results in the observation equations:

(6) $$f\left(x_{Ref},y_{Ref},z_{Ref}\right)+v\left(x_{Ref},y_{Ref},z_{Ref}\right)=r_{0}+r_{1}\cdot g\left(x_{Def},y_{Def},z_{Def}\right)$$

where $v(x_{Ref},y_{Ref},z_{Ref})$ = residuum

The first terms of the Taylor series expansion are used to linearize the observation equation, see Equation (7) where initial values of the parameters are included. They are marked with an additional zero subscript. Section 2.2 shows a way to obtain initial values for 3D LSM.

(7) $$\begin{array}{c} f\left(x_{Ref},y_{Ref},z_{Ref}\right)+v\left(x_{Ref},y_{Ref},z_{Ref}\right)\approx r_{0,0}+dr_{0}+\left(r_{1,0}+dr_{1}\right)\cdot g\left(x_{Def,0},y_{Def,0},z_{Def,0}\right)\\ +r_{1}\cdot \left(g_{x,0}\cdot dx_{Def}+g_{y,0}\cdot dy_{Def}+g_{z,0}\cdot dz_{Def}\right) \end{array}$$

where

$r_{0,0}=\;\mathrm{initial value of the brightness parameter}\;r_{0}$
$r_{1,0}=\;\mathrm{initial value of the contrast parameter}\;r_{1}$
$x_{Def,0}=\;x\;\mathrm{position (deformed state) computed with the initial values}\;a_{i,0}\mathrm{of}a_{i}$
$y_{Def,0}=\;y\;\mathrm{position (deformed state) computed with the initial values}\;b_{i,0}\mathrm{of}b_{i}$
$z_{Def,0}=\;z\;\mathrm{position (deformed state) computed with the initial values}\;c_{i,0}\mathrm{of}c_{i}$
$g_{x,0},g_{y,0},g_{z,0}=\;volume gradients at the initial positions\;x_{Def,0},y_{Def,0},z_{Def,0}$

The volume gradients of the gray values are written with their short forms in Equation (7). The full notation is:

(8) $$\begin{array}{cc} g_{x,0}= & \frac{\partial g(x_{Def},y_{Def},z_{Def})}{\partial x_{Def}}{|}_{x_{Def},y_{Def},z_{Def}=x_{Def,0},y_{Def,0},z_{Def,0}}\\ g_{y,0}= & \frac{\partial g(x_{Def},y_{Def},z_{Def})}{\partial y_{Def}}{|}_{x_{Def},y_{Def},z_{Def}=x_{Def,0},y_{Def,0},z_{Def,0}}\\ g_{z,0}= & \frac{\partial g(x_{Def},y_{Def},z_{Def})}{\partial z_{Def}}{|}_{x_{Def},y_{Def},z_{Def}=x_{Def,0},y_{Def,0},z_{Def,0}} \end{array}$$

There are different ways to obtain these gradients: One possibility is the computation of central differences $g_{x,i},g_{y,i},g_{z,i}$ (numerical differentiation, Equation (9)) at integer positions $x_{Def,i},y_{Def,i},z_{Def,i}$ ($x_{Def,i}\in N,y_{Def,i}\in N,z_{Def,i}\in N$) combined with a tri-linear interpolation in order to calculate the derivatives at the floating point values of $x_{Def},y_{Def}$ and $z_{Def}$.

(9) $$\begin{array}{cc} g_{x,i}\approx & 0.5\cdot (g\left(x_{Def,i}+1,y_{Def,i},z_{Def,i}\right)-g\left(x_{Def,i}-1,y_{Def,i},z_{Def,i}\right))\\ g_{y,i}\approx & 0.5\cdot (g\left(x_{Def,i},y_{Def,i}+1,z_{Def,i}\right)-g\left(x_{Def,i},y_{Def,i}-1,z_{Def,i}\right))\\ g_{z,i}\approx & 0.5\cdot (g\left(x_{Def,i},y_{Def,i},z_{Def,i}+1\right)-g\left(x_{Def,i},y_{Def,i},z_{Def,i}-1\right)) \end{array}$$

where

$g_{x,i}=\;derivative in\;x\;direction at integer positions$
$g_{y,i}=\;derivative in\;y\;direction at integer positions$
$g_{z,i}=\;derivative in\;z\;direction at integer positions$

Another approach is the use of tri-cubic spline interpolation to compute the derivatives [17] (derivatives of cubic polynomials). And a further possibility is the calculation of the derivatives in the reference volume as an approximation. The advantage of the last point is once-only computation in the iterative least-squares process.

Thus, the differentials $dx_{Def}$, $dy_{Def}$ and $dz_{Def}$ are:

(10) $$\begin{array}{cc} dx_{Def} & =\sum_{i=0}^{3}\frac{\partial x_{Def}}{\partial a_{i}}\cdot da_{i}=da_{0}+\tilde{x}\cdot da_{1}+\tilde{y}\cdot da_{2}+\tilde{z}\cdot da_{3}\\ dy_{Def} & =\sum_{i=0}^{3}\frac{\partial y_{Def}}{\partial b_{i}}\cdot db_{i}=db_{0}+\tilde{x}\cdot db_{1}+\tilde{y}\cdot db_{2}+\tilde{z}\cdot db_{3}\\ dz_{Def} & =\sum_{i=0}^{3}\frac{\partial z_{Def}}{\partial c_{i}}\cdot dc_{i}=dc_{0}+\tilde{x}\cdot dc_{1}+\tilde{y}\cdot dc_{2}+\tilde{z}\cdot dc_{3} \end{array}$$

where

${dx}_{Def}=\;\mathrm{differential of the}\;x\;\mathrm{coordinate}$
${dy}_{Def}=\;\mathrm{differential of the}\;y\;\mathrm{coordinate}$
${dz}_{Def}=\;\mathrm{differential of the}\;z\;\mathrm{coordinate}$

The vector of corrections to the unknowns is:

(11) $$dp={\left(\begin{array}{cccccccccccccc} da_{0}, & da_{1}, & da_{2}, & da_{3}, & db_{0}, & db_{1}, & db_{2}, & db_{3}, & dc_{0}, & dc_{1}, & dc_{2}, & dc_{3}, & dr_{0}, & dr_{1} \end{array}\right)}^{T}$$

The vector of unknowns results from the sum of the vector of the initial values $p_{0}$ and the vector of the corrections to the unknowns dp:

(12) $$p=p_{0}+dp$$

The linearized observation equations can be written in matrix notation:

(13) A·dp=l+v

where

**A** = Jacobian matrix
**l** = reduced observation vector
**v** = residual vector

The Jacobian matrix is built up as follows:

(14) $$A=\left(\begin{array}{cccc} A_{1} & A_{2} & A_{3} & A_{4} \end{array}\right)$$

where

(15) $$A_{1}=\left(\begin{array}{cccc} \begin{array}{c} \frac{\partial f}{\partial a_{0}}{|}_{p=p_{0}} \end{array} & \frac{\partial f}{\partial a_{1}}{|}_{p=p_{0}} & \frac{\partial f}{\partial a_{2}}{|}_{p=p_{0}} & \frac{\partial f}{\partial a_{3}}{|}_{p=p_{0}}\\ \vdots & \vdots & \vdots & \vdots \end{array}\right)=r_{1}\cdot \left(\begin{array}{cccc} g_{x,1} & g_{x,1}\cdot {\tilde{x}}_{1} & g_{x,1}\cdot {\tilde{y}}_{1} & g_{x,1}\cdot {\tilde{z}}_{1}\\ g_{x,2} & g_{x,2}\cdot {\tilde{x}}_{2} & g_{x,2}\cdot {\tilde{y}}_{2} & g_{x,2}\cdot {\tilde{z}}_{2}\\ \vdots & \vdots & \vdots & \vdots \\ g_{x,n} & g_{x,n}\cdot {\tilde{x}}_{n} & g_{x,n}\cdot {\tilde{y}}_{n} & g_{x,n}\cdot {\tilde{z}}_{n} \end{array}\right)$$

(16) $$A_{2}=\left(\begin{array}{cccc} \begin{array}{c} \frac{\partial f}{\partial b_{0}}{|}_{p=p_{0}} \end{array} & \frac{\partial f}{\partial b_{1}}{|}_{p=p_{0}} & \frac{\partial f}{\partial b_{2}}{|}_{p=p_{0}} & \frac{\partial f}{\partial b_{3}}{|}_{p=p_{0}}\\ \vdots & \vdots & \vdots & \vdots \end{array}\right)=r_{1}\cdot \left(\begin{array}{cccc} g_{y,1} & g_{y,1}\cdot {\tilde{x}}_{1} & g_{y,1}\cdot {\tilde{y}}_{1} & g_{y,1}\cdot {\tilde{z}}_{1}\\ g_{y,2} & g_{y,2}\cdot {\tilde{x}}_{2} & g_{y,2}\cdot {\tilde{y}}_{2} & g_{y,2}\cdot {\tilde{z}}_{2}\\ \vdots & \vdots & \vdots & \vdots \\ g_{y,n} & g_{y,n}\cdot {\tilde{x}}_{n} & g_{y,n}\cdot {\tilde{y}}_{n} & g_{y,n}\cdot {\tilde{z}}_{n} \end{array}\right)$$

(17) $$A_{3}=\left(\begin{array}{cccc} \begin{array}{c} \frac{\partial f}{\partial c_{0}}{|}_{p=p_{0}} \end{array} & \frac{\partial f}{\partial c_{1}}{|}_{p=p_{0}} & \frac{\partial f}{\partial c_{2}}{|}_{p=p_{0}} & \frac{\partial f}{\partial c_{3}}{|}_{p=p_{0}}\\ \vdots & \vdots & \vdots & \vdots \end{array}\right)=r_{1}\cdot \left(\begin{array}{cccc} g_{z,1} & g_{z,1}\cdot {\tilde{x}}_{1} & g_{z,1}\cdot {\tilde{y}}_{1} & g_{z,1}\cdot {\tilde{z}}_{1}\\ g_{z,2} & g_{z,2}\cdot {\tilde{x}}_{2} & g_{z,2}\cdot {\tilde{y}}_{2} & g_{z,2}\cdot {\tilde{z}}_{2}\\ \vdots & \vdots & \vdots & \vdots \\ g_{z,n} & g_{z,n}\cdot {\tilde{x}}_{n} & g_{z,n}\cdot {\tilde{y}}_{n} & g_{z,n}\cdot {\tilde{z}}_{n} \end{array}\right)$$

(18) $$A_{4}=\begin{array}{c} \left(\begin{array}{cc} \frac{\partial f}{\partial r_{0}}{|}_{p=p_{0}} & \frac{\partial f}{\partial r_{1}}{|}_{p=p_{0}}\\ \vdots & \vdots \end{array}\right)=\left(\begin{array}{cc} 1 & g_{1}\\ 1 & g_{2}\\ \vdots & \vdots \\ 1 & g_{n} \end{array}\right) \end{array}$$

The reduced observation vector l is:

(19) $$l=\left(\begin{array}{c} f_{1}-r_{0}-r_{1}\cdot g_{1}\\ f_{2}-r_{0}-r_{1}\cdot g_{2}\\ \vdots \\ f_{n}-r_{0}-r_{1}\cdot g_{n} \end{array}\right)$$

In the Gauss-Markov model, the weighted sum of the squared residuals is minimized:

(20) $$v^{T}\cdot W\cdot v\to \underset{dp}{\min}$$

where W = matrix of weights

In the following, only the case of equally weighted observations (W=I, I: identity matrix) is considered, see Equation (21).

(21) $$v^{T}\cdot v\to \underset{dp}{\min}$$

The solution of this problem can be obtained using the normal equations:

(22) $$A^{T}\cdot A\cdot dp=A^{T}\cdot l$$

As the computation of the normal matrix $A^{T}\cdot A$ and the right hand side $A^{T}\cdot l$ are done directly, it is not necessary to build up and store the whole Jacobian matrix A and the reduced observation vector l. The detailed computation process is shown in Section 2.3.

After setting up the matrices $A^{T}\cdot A$ and $A^{T}\cdot l$, Equation (22) is solved for dp and the initial values of the unknowns are updated:

(23) $$p=p_{0}+dp$$

The steps of the interpolation of the gray values and their derivatives as well as the computation of $A^{T}\cdot A$ and $A^{T}\cdot l$, the determination of dp and the update of the unknowns are repeated until the process converges.

After the iterative adjustment routine, the residuals are determined by rearranging Equation (6). The gray values $g_{i}$ are obtained by applying Equation (3) with the updated affine parameters and tri-linear (or tri-cubic) interpolation. The individual residual $v_{i}$ is:

(24) $$v_{i}=r_{0}+r_{1}\cdot g_{i}\left(x_{Def},y_{Def},z_{Def}\right)-f_{i}\left(x_{Ref},y_{Ref},z_{Ref}\right)$$

where $v_{i}$ = individual residual

The standard deviation of the unit weight is:

(25) $$s_{0}=\sqrt{\frac{\sum_{i=1}^{n}v_{i}^{2}}{n-u}}$$

where

*s*_0_ = standard deviation of the unit weight
*n* = number of voxel of the cuboid
*n* = number of unknowns

### 2.2. Initial Values

The model in Equation (2) is not linear and if the movements between the volume data sets may exceed the dimensions of the cuboid, initial values have to be obtained for the 3D LSM algorithm.

Often, mainly translations and only small rotations occur between different epochs of measurements. In these cases, 3D cross-correlation can be used to obtain initial shifts [10]. An overview of the optimized algorithm can be found in Appendix A.

### 2.3. Direct Computation of the Normal Equations

As mentioned above, the normal matrix ($A^{T}\cdot A$) and the right hand side vector ($A^{T}\cdot l$) are computed directly, so that building up the Jacobian matrix A as well as the reduced observation vector l and the matrix multiplications can be avoided in order to gain efficiency and save memory. To compute the terms of the normal equations, the dot products of the columns of the Jacobian matrix (Equation (14)) as well as the dot products with the reduced observation vector are calculated (each combination of columns). Equation (26) shows the normal matrix that is organized in submatrices. $G_{11,i}$, $G_{12,i}$, $G_{13,i}$, $G_{22,i}$, $G_{23,i}$, $G_{33,i}$ and $G_{44,i}$ as well as the whole normal matrix are symmetric matrices and only the upper triangular matrices are shown. In Equation (28), the right hand side of the normal equations is depicted.

(26) $$A^{T}\cdot A=\left(\begin{array}{cccc} A_{1}^{T}\cdot A_{1} & A_{1}^{T}\cdot A_{2} & A_{1}^{T}\cdot A_{3} & A_{1}^{T}\cdot A_{4}\\ \; & A_{2}^{T}\cdot A_{2} & A_{2}^{T}\cdot A_{3} & A_{2}^{T}\cdot A_{4}\\ & \; & A_{3}^{T}\cdot A_{3} & A_{3}^{T}\cdot A_{4}\\ & \; & & A_{4}^{T}\cdot A_{4} \end{array}\right)=\left(\begin{array}{cccc} r_{1}^{2}\cdot \sum_{i=1}^{n}G_{11,i} & r_{1}^{2}\cdot \sum_{i=1}^{n}G_{12,i}\; & r_{1}^{2}\cdot \sum_{i=1}^{n}G_{13,i} & r_{1}\cdot \sum_{i=1}^{n}G_{14,i}\\ \; & r_{1}^{2}\cdot \sum_{i=1}^{n}G_{22,i}\; & r_{1}^{2}\cdot \sum_{i=1}^{n}G_{23,i} & r_{1}\cdot \sum_{i=1}^{n}G_{24,i}\\ \; & \; & r_{1}^{2}\cdot \sum_{i=1}^{n}G_{33,i} & r_{1}\cdot \sum_{i=1}^{n}G_{34,i}\\ \; & \; & & \sum_{i=1}^{n}G_{44,i} \end{array}\right)$$

where

(27) $$G_{11,i}=\left(\begin{array}{cccc} \begin{array}{c} g_{x,i}^{2} \end{array} & g_{x,i}^{2}\cdot {\tilde{x}}_{i} & g_{x,i}^{2}\cdot {\tilde{y}}_{i} & g_{x,i}^{2}\cdot {\tilde{z}}_{i}\\ \; & g_{x,i}^{2}\cdot {\tilde{x}}_{i}^{2} & g_{x,i}^{2}\cdot {\tilde{x}}_{i}\cdot {\tilde{y}}_{i} & g_{x,i}^{2}\cdot {\tilde{x}}_{i}\cdot {\tilde{z}}_{i}\\ \; & \; & g_{x,i}^{2}\cdot {\tilde{y}}_{i}^{2} & g_{x,i}^{2}\cdot {\tilde{y}}_{i}\cdot {\tilde{z}}_{i}\\ \; & \; & & g_{x,i}^{2}\cdot {\tilde{z}}_{i}^{2} \end{array}\right),\;G_{12,i}=\left(\begin{array}{cccc} \begin{array}{c} g_{x,i}\cdot g_{y,i} \end{array} & g_{x,i}\cdot gyx_{i}\cdot {\tilde{x}}_{i} & g_{x,i}\cdot gyy_{i}\cdot {\tilde{y}}_{i} & g_{x,i}\cdot gyz_{i}\cdot {\tilde{z}}_{i}\\ \; & gxx_{i}\cdot gxy_{i}\cdot {\tilde{x}}_{i}^{2} & gxx_{i}\cdot gyy_{i}\cdot {\tilde{x}}_{i}\cdot {\tilde{y}}_{i} & gx_{i}\cdot gy_{i}\cdot {\tilde{x}}_{i}\cdot {\tilde{z}}_{i}\\ \; & \; & gx_{i}\cdot gy_{i}\cdot {\tilde{y}}_{i}^{2} & gx_{i}\cdot gy_{i}\cdot {\tilde{y}}_{i}\cdot {\tilde{z}}_{i}\\ \; & \; & & gx_{i}\cdot gy_{i}\cdot {\tilde{z}}_{i}^{2} \end{array}\right),\;\;G_{22,i}=\left(\begin{array}{cccc} \begin{array}{c} g_{y,i}^{2} \end{array} & g_{y,i}^{2}\cdot {\tilde{x}}_{i} & g_{y,i}^{2}\cdot {\tilde{y}}_{i} & g_{y,i}^{2}\cdot {\tilde{z}}_{i}\\ \; & g_{y,i}^{2}\cdot {\tilde{x}}_{i}^{2} & g_{y,i}^{2}\cdot {\tilde{x}}_{i}\cdot {\tilde{y}}_{i} & g_{y,i}^{2}\cdot {\tilde{x}}_{i}\cdot {\tilde{z}}_{i}\\ \; & \; & g_{y,i}^{2}\cdot {\tilde{y}}_{i} & g_{y,i}^{2}\cdot {\tilde{y}}_{i}\cdot {\tilde{z}}_{i}\\ \; & \; & & g_{y,i}^{2}\cdot {\tilde{z}}_{i}^{2} \end{array}\right),\;G_{13,i}=\left(\begin{array}{cccc} \begin{array}{c} g_{x,i}\cdot g_{z,i} \end{array} & g_{x,i}\cdot gzx_{i}\cdot {\tilde{x}}_{i} & g_{x,i}\cdot gzy_{i}\cdot {\tilde{y}}_{i} & g_{x,i}\cdot gzz_{i}\cdot {\tilde{z}}_{i}\\ \; & g_{x,i}\cdot g_{z,i}\cdot {\tilde{x}}_{i}^{2} & g_{x,i}\cdot g_{z,i}\cdot {\tilde{x}}_{i}\cdot {\tilde{y}}_{i} & g_{x,i}\cdot g_{z,i}\cdot {\tilde{x}}_{i}\cdot {\tilde{z}}_{i}\\ \; & \; & g_{x,i}\cdot g_{z,i}\cdot {\tilde{y}}_{i}^{2} & g_{x,i}\cdot g_{z,i}\cdot {\tilde{y}}_{i}\cdot {\tilde{z}}_{i}\\ \; & \; & & g_{x,i}\cdot g_{z,i}\cdot {\tilde{z}}_{i}^{2} \end{array}\right),\;\;G_{23,i}=\left(\begin{array}{cccc} \begin{array}{c} g_{y,i}\cdot g_{z,i} \end{array} & g_{y,i}\cdot g_{z,i}\cdot \cdot {\tilde{x}}_{i} & g_{y,i}\cdot g_{z,i}\cdot {\tilde{y}}_{i} & g_{y,i}\cdot g_{z,i}\cdot {\tilde{z}}_{i}\\ \; & g_{y,i}\cdot g_{z,i}\cdot {\tilde{x}}_{i}^{2} & g_{y,i}\cdot g_{z,i}\cdot {\tilde{x}}_{i}\cdot {\tilde{y}}_{i} & g_{y,i}\cdot g_{z,i}\cdot {\tilde{x}}_{i}\cdot {\tilde{z}}_{i}\\ \; & \; & g_{y,i}\cdot g_{z,i}\cdot {\tilde{y}}_{i}^{2} & g_{y,i}\cdot g_{z,i}\cdot {\tilde{y}}_{i}\cdot {\tilde{z}}_{i}\\ \; & \; & & g_{y,i}\cdot g_{z,i}\cdot {\tilde{z}}_{i}^{2} \end{array}\right),\;G_{33,i}=\left(\begin{array}{cccc} \begin{array}{c} g_{z,i}^{2} \end{array} & g_{z,i}^{2}\cdot {\tilde{y}}_{i} & g_{z,i}^{2}\cdot {\tilde{y}}_{i} & g_{z,i}^{2}\cdot {\tilde{z}}_{i}\\ \; & g_{z,i}^{2}\cdot {\tilde{x}}_{i}^{2} & g_{z,i}^{2}\cdot {\tilde{x}}_{i}\cdot {\tilde{y}}_{i} & g_{z,i}^{2}\cdot {\tilde{x}}_{i}\cdot {\tilde{z}}_{i}\\ \; & \; & g_{z,i}^{2}\cdot {\tilde{y}}_{i}^{2} & g_{z,i}^{2}\cdot {\tilde{y}}_{i}\cdot {\tilde{z}}_{i}\\ \; & \; & & g_{z,i}^{2}\cdot {\tilde{z}}_{i}^{2} \end{array}\right),\;\;G_{14,i}=\left(\begin{array}{cc} \begin{array}{c} g_{x,i} \end{array} & g_{i}\cdot g_{x,i}\\ g_{x,i}\cdot {\tilde{x}}_{i} & g_{i}\cdot g_{x,i}\cdot {\tilde{x}}_{i}\\ g_{x,i}\cdot {\tilde{x}}_{i} & g_{i}\cdot g_{x,i}\cdot {\tilde{x}}_{i}\\ g_{x,i}\cdot {\tilde{z}}_{i} & g_{i}\cdot g_{x,i}\cdot {\tilde{z}}_{i} \end{array}\right),\;G_{24,i}=\left(\begin{array}{cc} \begin{array}{c} g_{y,i} \end{array} & g_{i}\cdot g_{y,i}\\ g_{y,i}\cdot {\tilde{x}}_{i} & g_{i}\cdot g_{y,i}\cdot {\tilde{x}}_{i}\\ g_{y,i}\cdot {\tilde{y}}_{i} & g_{i}\cdot g_{y,i}\cdot {\tilde{y}}_{i}\\ g_{y,i}\cdot {\tilde{z}}_{i} & g_{i}\cdot g_{y,i}\cdot {\tilde{z}}_{i} \end{array}\right),\;G_{34,i}=\left(\begin{array}{cc} \begin{array}{c} g_{z,i} \end{array} & g_{i}\cdot g_{z,i}\\ g_{z,i}\cdot {\tilde{x}}_{i} & g_{i}\cdot g_{z,i}\cdot {\tilde{x}}_{i}\\ g_{z,i}\cdot {\tilde{y}}_{i} & g_{i}\cdot g_{z,i}\cdot {\tilde{y}}_{i}\\ g_{z,i}\cdot {\tilde{z}}_{i} & g_{i}\cdot g_{z,i}\cdot {\tilde{z}}_{i} \end{array}\right),\;G_{44,i}=\left(\begin{array}{cc} 1 & g_{i}\\ & g_{i}^{2} \end{array}\right)$$

(28) $$A^{T}\cdot l=\left(\begin{array}{c} A_{1}^{T}\cdot l\\ A_{2}^{T}\cdot l\\ A_{3}^{T}\cdot l\\ A_{4}^{T}\cdot l \end{array}\right)=\left(\begin{array}{c} r_{1}\cdot \sum_{i=1}^{n}f_{i}\cdot g_{x,i}-r_{0}\cdot r_{1}\cdot \sum_{i=1}^{n}g_{x,i}-r_{1}^{2}\cdot \sum_{i=1}^{n}g_{i}\cdot g_{x,i}\\ r_{1}\cdot \sum_{i=1}^{n}f_{i}\cdot g_{x,i}\cdot {\tilde{x}}_{i}-r_{0}\cdot r_{1}\cdot \sum_{i=1}^{n}g_{x,i}\cdot {\tilde{x}}_{i}-r_{1}^{2}\cdot \sum_{i=1}^{n}g_{i}\cdot g_{x,i}\cdot {\tilde{x}}_{i}\\ r_{1}\cdot \sum_{i=1}^{n}f_{i}\cdot g_{x,i}\cdot {\tilde{y}}_{i}-r_{0}\cdot r_{1}\cdot \sum_{i=1}^{n}g_{x,i}\cdot {\tilde{y}}_{i}-r_{1}^{2}\cdot \sum_{i=1}^{n}g_{i}\cdot g_{x,i}\cdot {\tilde{y}}_{i}\\ r_{1}\cdot \sum_{i=1}^{n}f_{i}\cdot g_{x,i}\cdot {\tilde{z}}_{i}-r_{0}\cdot r_{1}\cdot \sum_{i=1}^{n}g_{x,i}\cdot {\tilde{z}}_{i}-r_{1}^{2}\cdot \sum_{i=1}^{n}g_{i}\cdot g_{x,i}\cdot {\tilde{z}}_{i}\\ r_{1}\cdot \sum_{i=1}^{n}f_{i}\cdot g_{y,i}-r_{0}\cdot r_{1}\cdot \sum_{i=1}^{n}g_{y,i}-r_{1}^{2}\cdot \sum_{i=1}^{n}g_{i}\cdot g_{y,i}\\ r_{1}\cdot \sum_{i=1}^{n}f_{i}\cdot g_{y,i}\cdot {\tilde{x}}_{i}-r_{0}\cdot r_{1}\cdot \sum_{i=1}^{n}g_{y,i}\cdot {\tilde{x}}_{i}-r_{1}^{2}\cdot \sum_{i=1}^{n}g_{i}\cdot g_{y,i}\cdot {\tilde{x}}_{i}\\ r_{1}\cdot \sum_{i=1}^{n}f_{i}\cdot g_{y,i}\cdot {\tilde{y}}_{i}-r_{0}\cdot r_{1}\cdot \sum_{i=1}^{n}g_{y,i}\cdot {\tilde{y}}_{i}-r_{1}^{2}\cdot \sum_{i=1}^{n}g_{i}\cdot g_{y,i}\cdot {\tilde{y}}_{i}\\ r_{1}\cdot \sum_{i=1}^{n}f_{i}\cdot g_{y,i}\cdot {\tilde{z}}_{i}-r_{0}\cdot r_{1}\cdot \sum_{i=1}^{n}g_{y,i}\cdot {\tilde{z}}_{i}-r_{1}^{2}\cdot \sum_{i=1}^{n}g_{i}\cdot g_{y,i}\cdot {\tilde{z}}_{i}\\ r_{1}\cdot \sum_{i=1}^{n}f_{i}\cdot g_{z,i}-r_{0}\cdot r_{1}\cdot \sum_{i=1}^{n}g_{z,i}-r_{1}^{2}\cdot \sum_{i=1}^{n}g_{i}\cdot g_{z,i}\\ r_{1}\cdot \sum_{i=1}^{n}f_{i}\cdot g_{z,i}\cdot {\tilde{x}}_{i}-r_{0}\cdot r_{1}\cdot \sum_{i=1}^{n}g_{z,i}\cdot {\tilde{x}}_{i}-r_{1}^{2}\cdot \sum_{i=1}^{n}g_{i}\cdot g_{z,i}\cdot {\tilde{x}}_{i}\\ r_{1}\cdot \sum_{i=1}^{n}f_{i}\cdot g_{z,i}\cdot {\tilde{y}}_{i}-r_{0}\cdot r_{1}\cdot \sum_{i=1}^{n}g_{z,i}\cdot {\tilde{y}}_{i}-r_{1}^{2}\cdot \sum_{i=1}^{n}g_{i}\cdot g_{z,i}\cdot {\tilde{y}}_{i}\\ r_{1}\cdot \sum_{i=1}^{n}f_{i}\cdot g_{z,i}\cdot {\tilde{z}}_{i}-r_{0}\cdot r_{1}\cdot \sum_{i=1}^{n}g_{z,i}\cdot {\tilde{z}}_{i}-r_{1}^{2}\cdot \sum_{i=1}^{n}g_{i}\cdot g_{z,i}\cdot {\tilde{z}}_{i}\\ \sum_{i=1}^{n}f_{i}-r_{0}\cdot n-r_{1}\cdot \sum_{i=1}^{n}g_{i}\\ \sum_{i=1}^{n}f_{i}\cdot g_{i}-r_{0}\cdot \sum_{i=1}^{n}g_{i}-r_{1}\cdot \sum_{i=1}^{n}g_{i}^{2} \end{array}\right)$$

In fact, the geometric and radiometric parameters are usually independent on each other and are therefore often solved independently in LSM. If only the affine parameters are unknowns and the radiometric parameters are excluded from the model, the last two columns and last two rows of $A^{T}\cdot A$ as well as the last two values of $A^{T}\cdot l$ are omitted. Appendix B and Appendix C consider the two cases concerning this point.

For each voxel, the following terms are computed and saved in temporal variables because they are used several times:

(29) $$\begin{array}{c} gxx_{i}=g_{x,i}\cdot {\tilde{x}}_{i}\\ gxy_{i}=g_{x,i}\cdot {\tilde{y}}_{i}\\ gxz_{i}=g_{x,i}\cdot {\tilde{z}}_{i}\\ gyx_{i}=g_{y,i}\cdot {\tilde{x}}_{i}\\ gyy_{i}=g_{y,i}\cdot {\tilde{y}}_{i}\\ gyz_{i}=g_{y,i}\cdot {\tilde{z}}_{i}\\ gzx_{i}=g_{z,i}\cdot {\tilde{x}}_{i}\\ gzy_{i}=g_{z,i}\cdot {\tilde{y}}_{i}\\ gzz_{i}=g_{z,i}\cdot {\tilde{z}}_{i} \end{array}$$

Substituting the terms of Equation (29), the submatrices of the normal matrix and the right hand sight are shown in Equations (30) and (31). This reduces the number of computation steps, too.

(30) $$G_{11,i}=\left(\begin{array}{cccc} \begin{array}{c} g_{x,i}^{2} \end{array} & g_{x,i}\cdot gxx_{i} & g_{x,i}\cdot gxy_{i} & g_{x,i}\cdot gxz_{i}\\ \; & gxx_{i}^{2} & gxx_{i}\cdot gxy_{i} & gxx_{i}\cdot gxz_{i}\\ \; & \; & gxy_{i}^{2} & gxy_{i}\cdot gxz_{i}\\ \; & \; & & gxz_{i}^{2} \end{array}\right),\;G_{12,i}=\left(\begin{array}{cccc} \begin{array}{c} g_{x,i}\cdot g_{y,i} \end{array} & g_{x,i}\cdot gyx_{i} & g_{x,i}\cdot gyy_{i} & g_{x,i}\cdot gyz_{i}\\ \; & gxx_{i}\cdot gyx_{i} & gxx_{i}\cdot gyy_{i} & gxx_{i}\cdot gyz_{i}\\ \; & \; & gxy_{i}\cdot gyy_{i} & gxy_{i}\cdot gyz_{i}\\ \; & \; & & gxz_{i}\cdot gyz_{i} \end{array}\right),\;\;G_{22,i}=\left(\begin{array}{cccc} \begin{array}{c} g_{y,i}^{2} \end{array} & g_{y,i}\cdot gyx_{i} & g_{y,i}\cdot gyy_{i} & g_{y,i}\cdot gyz_{i}\\ \; & gyx_{i}^{2} & gyx_{i}\cdot gyy_{i} & gyx_{i}\cdot gyz_{i}\\ \; & \; & gyy_{i}^{2} & gyy_{i}\cdot gyz_{i}\\ \; & \; & & gyz_{i}^{2} \end{array}\right),\;G_{13,i}=\left(\begin{array}{cccc} \begin{array}{c} g_{x,i}\cdot g_{z,i} \end{array} & g_{x,i}\cdot gzx_{i} & g_{x,i}\cdot gzy_{i} & g_{x,i}\cdot gzz_{i}\\ \; & gxx_{i}\cdot gzx_{i} & gxx_{i}\cdot gzy_{i} & gxx_{i}\cdot gzz_{i}\\ \; & \; & gxy_{i}\cdot gzy_{i} & gxy_{i}\cdot gzz_{i}\\ \; & \; & & gxz_{i}\cdot gzz_{i} \end{array}\right),\;\;G_{23,i}=\left(\begin{array}{cccc} \begin{array}{c} g_{y,i}\cdot g_{z,i} \end{array} & g_{y,i}\cdot gzx_{i} & g_{y,i}\cdot gzy_{i} & g_{y,i}\cdot gzz_{i}\\ \; & gyx_{i}\cdot gzx_{i} & gyx_{i}\cdot gzy_{i} & gyx_{i}\cdot gzz_{i}\\ \; & \; & gyy_{i}\cdot gzy_{i} & gyy_{i}\cdot gzz_{i}\\ \; & \; & & gyz_{i}\cdot gzz_{i} \end{array}\right),\;G_{33,i}=\left(\begin{array}{cccc} \begin{array}{c} g_{z,i}^{2} \end{array} & g_{z,i}\cdot gzx_{i} & g_{z,i}\cdot gzy_{i} & g_{z,i}\cdot gzz_{i}\\ \; & gzx_{i}^{2} & gzx_{i}\cdot gzy_{i} & gzx_{i}\cdot gzz_{i}\\ \; & \; & gzy_{i}^{2} & gzy_{i}\cdot gzz_{i}\\ \; & \; & & gzz_{i}^{2} \end{array}\right),\;\;G_{14,i}=\left(\begin{array}{cc} \begin{array}{c} g_{x,i} \end{array} & g_{i}\cdot g_{x,i}\\ gxx_{i} & g_{i}\cdot gxx_{i}\\ gxy_{i} & g_{i}\cdot gxy_{i}\\ gxz_{i} & g_{i}\cdot gxz_{i} \end{array}\right),\;G_{24,i}=\left(\begin{array}{cc} \begin{array}{c} g_{y,i} \end{array} & g_{i}\cdot g_{y,i}\\ gyx_{i} & g_{i}\cdot gyx_{i}\\ gyy_{i} & g_{i}\cdot gyy_{i}\\ gyz_{i} & g_{i}\cdot gyz_{i} \end{array}\right),\;G_{34,i}=\left(\begin{array}{cc} \begin{array}{c} g_{z,i} \end{array} & g_{i}\cdot g_{z,i}\\ gzx_{i} & g_{i}\cdot gzx_{i}\\ gzy_{i} & g_{i}\cdot gzy_{i}\\ gzz_{i} & g_{i}\cdot gzz_{i} \end{array}\right),\;G_{44,i}=\left(\begin{array}{cc} 1 & g_{i}\\ & g_{i}^{2} \end{array}\right)$$

(31) $$A^{T}\cdot l=\left(\begin{array}{c} r_{1}\cdot \sum_{i=1}^{n}f_{i}\cdot g_{x,i}-r_{0}\cdot r_{1}\cdot \sum_{i=1}^{n}g_{x,i}-r_{1}^{2}\cdot \sum_{i=1}^{n}g_{i}\cdot g_{x,i}\\ r_{1}\cdot \sum_{i=1}^{n}f_{i}\cdot gxx_{i}-r_{0}\cdot r_{1}\cdot \sum_{i=1}^{n}gxx_{i}-r_{1}^{2}\cdot \sum_{i=1}^{n}g_{i}\cdot gxx_{i}\\ r_{1}\cdot \sum_{i=1}^{n}f_{i}\cdot gxy_{i}-r_{0}\cdot r_{1}\cdot \sum_{i=1}^{n}gxy_{i}-r_{1}^{2}\cdot \sum_{i=1}^{n}g_{i}\cdot gxy_{i}\\ r_{1}\cdot \sum_{i=1}^{n}f_{i}\cdot gxz_{i}-r_{0}\cdot r_{1}\cdot \sum_{i=1}^{n}gxz_{i}-r_{1}^{2}\cdot \sum_{i=1}^{n}g_{i}\cdot gxz_{i}\\ r_{1}\cdot \sum_{i=1}^{n}f_{i}\cdot g_{y,i}-r_{0}\cdot r_{1}\cdot \sum_{i=1}^{n}g_{y,i}-r_{1}^{2}\cdot \sum_{i=1}^{n}g_{i}\cdot g_{y,i}\\ r_{1}\cdot \sum_{i=1}^{n}f_{i}\cdot gyx_{i}-r_{0}\cdot r_{1}\cdot \sum_{i=1}^{n}gyx_{i}-r_{1}^{2}\cdot \sum_{i=1}^{n}g_{i}\cdot gyx_{i}\\ r_{1}\cdot \sum_{i=1}^{n}f_{i}\cdot gyy_{i}-r_{0}\cdot r_{1}\cdot \sum_{i=1}^{n}gyy_{i}-r_{1}^{2}\cdot \sum_{i=1}^{n}g_{i}\cdot gyy_{i}\\ r_{1}\cdot \sum_{i=1}^{n}f_{i}\cdot gyz_{i}-r_{0}\cdot r_{1}\cdot \sum_{i=1}^{n}gyz_{i}-r_{1}^{2}\cdot \sum_{i=1}^{n}g_{i}\cdot gyz_{i}\\ r_{1}\cdot \sum_{i=1}^{n}f_{i}\cdot g_{z,i}-r_{0}\cdot r_{1}\cdot \sum_{i=1}^{n}g_{z,i}-r_{1}^{2}\cdot \sum_{i=1}^{n}g_{i}\cdot g_{z,i}\\ r_{1}\cdot \sum_{i=1}^{n}f_{i}\cdot gzx_{i}-r_{0}\cdot r_{1}\cdot \sum_{i=1}^{n}gzx_{i}-r_{1}^{2}\cdot \sum_{i=1}^{n}g_{i}\cdot gzx_{i}\\ r_{1}\cdot \sum_{i=1}^{n}f_{i}\cdot gzy_{i}-r_{0}\cdot r_{1}\cdot \sum_{i=1}^{n}gzy_{i}-r_{1}^{2}\cdot \sum_{i=1}^{n}g_{i}\cdot gzy_{i}\\ r_{1}\cdot \sum_{i=1}^{n}f_{i}\cdot gzz_{i}-r_{0}\cdot r_{1}\cdot \sum_{i=1}^{n}gzz_{i}-r_{1}^{2}\cdot \sum_{i=1}^{n}g_{i}\cdot gzz_{i}\\ \sum_{i=1}^{n}f_{i}-r_{0}\cdot n-r_{1}\cdot \sum_{i=1}^{n}g_{i}\\ \sum_{i=1}^{n}f_{i}\cdot g_{i}-r_{0}\cdot \sum_{i=1}^{n}g_{i}-r_{1}\cdot \sum_{i=1}^{n}g_{i}^{2} \end{array}\right)$$

### 2.4. Computational Effort

The computational costs are compared between the direct computation and the standard way that includes building up A and l as well as the matrix multiplications ($A^{T}\cdot A$ and $A^{T}\cdot l$). The detailed determination of the number of multiplication steps and addition steps is shown in the following.

#### 2.4.1. Computational Costs of the Direct Method

A total of 100 terms have to be computed to form the normal equations directly, see Equation (32). This results from 60 terms of the matrices $G_{11,i}$, $G_{12,i}$, $G_{13,i}$, $G_{22,i}$, $G_{23,i}$, $G_{33,i}$ and 26 terms of the matrices $G_{14,i}$, $G_{24,i}$, $G_{34,i}$, $G_{44,i}$ as well as 14 further terms of $A^{T}\cdot l$. 26 terms (sums) of $A^{T}\cdot l$ are already computed in $G_{14,i}$, $G_{24,i}$, $G_{34,i}$ and $G_{44,i}$.

(32) $$\begin{array}{c} \begin{array}{c} \sum_{i=1}^{n}g_{x,i}^{2},\sum_{i=1}^{n}g_{x,i}\cdot gxx_{i},\sum_{i=1}^{n}g_{x,i}\cdot gxy_{i},\sum_{i=1}^{n}g_{x,i}\cdot gxz_{i},\sum_{i=1}^{n}gxx_{i}^{2},\sum_{i=1}^{n}gxx_{i}\cdot gxy_{i},\sum_{i=1}^{n}gxx_{i}\cdot gxz_{i},\sum_{i=1}^{n}gxy_{i}^{2},\sum_{i=1}^{n}gxy_{i}\cdot gxz_{i},\sum_{i=1}^{n}gxz_{i}^{2}, \end{array}\\ \begin{array}{c} \sum_{i=1}^{n}g_{x,i}\cdot g_{y,i},\sum_{i=1}^{n}g_{x,i}\cdot gyx_{i},\sum_{i=1}^{n}g_{x,i}\cdot gyy_{i},\sum_{i=1}^{n}g_{x,i}\cdot gyz_{i},\sum_{i=1}^{n}gxx_{i}\cdot gyx_{i},\sum_{i=1}^{n}gxx_{i}\cdot gyy_{i},\sum_{i=1}^{n}gxx_{i}\cdot gyz_{i},\sum_{i=1}^{n}gxy_{i}\cdot gyy_{i},\sum_{i=1}^{n}gxy_{i}\cdot gyz_{i},\sum_{i=1}^{n}gxz_{i}\cdot gyz_{i}, \end{array}\\ \begin{array}{c} \sum_{i=1}^{n}g_{x,i}\cdot g_{z,i},\sum_{i=1}^{n}g_{x,i}\cdot gzx_{i},\sum_{i=1}^{n}g_{x,i}\cdot gzy_{i},\sum_{i=1}^{n}g_{x,i}\cdot gzz_{i},\sum_{i=1}^{n}gxx_{i}\cdot gzx_{i},\sum_{i=1}^{n}gxx_{i}\cdot gzy_{i},\sum_{i=1}^{n}gxx_{i}\cdot gzz_{i},\sum_{i=1}^{n}gxy_{i}\cdot gzy_{i},\sum_{i=1}^{n}gxy_{i}\cdot gzz_{i},\sum_{i=1}^{n}gxz_{i}\cdot gzz_{i}, \end{array}\\ \begin{array}{c} \sum_{i=1}^{n}g_{x,i},\sum_{i=1}^{n}g_{i}\cdot g_{x,i},\sum_{i=1}^{n}gxx_{i},\sum_{i=1}^{n}g_{i}\cdot gxx_{i},\sum_{i=1}^{n}gxy_{i},\sum_{i=1}^{n}g_{i}\cdot gxy_{i},\sum_{i=1}^{n}gxz_{i},\sum_{i=1}^{n}g_{i}\cdot gxz_{i}, \end{array}\\ \begin{array}{c} \sum_{i=1}^{n}g_{y,i}^{2},\sum_{i=1}^{n}g_{y,i}\cdot gyx_{i},\sum_{i=1}^{n}g_{y,i}\cdot gyy_{i},\sum_{i=1}^{n}g_{y,i}\cdot gyz_{i},\sum_{i=1}^{n}gyx_{i}^{2},\sum_{i=1}^{n}gyx_{i}\cdot gyy_{i},\sum_{i=1}^{n}gyx_{i}\cdot gyz_{i},\sum_{i=1}^{n}gyy_{i}^{2},\sum_{i=1}^{n}gyy_{i}\cdot gyz_{i},\sum_{i=1}^{n}gyz_{i}^{2}, \end{array}\\ \begin{array}{c} \sum_{i=1}^{n}g_{y,i}\cdot g_{z,i},\sum_{i=1}^{n}g_{y,i}\cdot gzx_{i},\sum_{i=1}^{n}g_{y,i}\cdot gzy_{i},\sum_{i=1}^{n}g_{y,i}\cdot gzz_{i},\sum_{i=1}^{n}gyx_{i}\cdot gzx_{i},\sum_{i=1}^{n}gyx_{i}\cdot gzy_{i},\sum_{i=1}^{n}gyx_{i}\cdot gzz_{i},\sum_{i=1}^{n}gyy_{i}\cdot gzy_{i},\sum_{i=1}^{n}gyy_{i}\cdot gzz_{i},\sum_{i=1}^{n}gyz_{i}\cdot gzz_{i}, \end{array}\\ \begin{array}{c} \sum_{i=1}^{n}g_{y,i},\sum_{i=1}^{n}g_{i}\cdot g_{y,i},\sum_{i=1}^{n}gyx_{i},\sum_{i=1}^{n}g_{i}\cdot gyx_{i},\sum_{i=1}^{n}gyy_{i},\sum_{i=1}^{n}g_{i}\cdot gyy_{i},\sum_{i=1}^{n}gyz_{i},\sum_{i=1}^{n}g_{i}\cdot gyz_{i}, \end{array}\\ \begin{array}{c} \sum_{i=1}^{n}g_{z,i}^{2},\sum_{i=1}^{n}g_{z,i}\cdot gzx_{i},\sum_{i=1}^{n}g_{z,i}\cdot gzy_{i},\sum_{i=1}^{n}g_{z,i}\cdot gzz_{i},\sum_{i=1}^{n}gzx_{i}^{2},\sum_{i=1}^{n}gzx_{i}\cdot gzy_{i},\sum_{i=1}^{n}gzx_{i}\cdot gzz_{i},\sum_{i=1}^{n}gzy_{i}^{2},\sum_{i=1}^{n}gzy_{i}\cdot gzz_{i},\sum_{i=1}^{n}gzz_{i}^{2}, \end{array}\\ \begin{array}{c} \sum_{i=1}^{n}g_{z,i},\sum_{i=1}^{n}g_{i}\cdot g_{z,i},\sum_{i=1}^{n}gzx_{i},\sum_{i=1}^{n}g_{i}\cdot gzx_{i},\sum_{i=1}^{n}gzy_{i},\sum_{i=1}^{n}g_{i}\cdot gzy_{i},\sum_{i=1}^{n}gzz_{i},\sum_{i=1}^{n}g_{i}\cdot gzz_{i}, \end{array}\\ \begin{array}{c} \sum_{i=1}^{n}g_{i},\sum_{i=1}^{n}g_{i}^{2},\sum_{i=1}^{n}f_{i}\cdot g_{x,i},\sum_{i=1}^{n}f_{i}\cdot gxx_{i},\sum_{i=1}^{n}f_{i}\cdot gxy_{i},\sum_{i=1}^{n}f_{i}\cdot gxz_{i},\sum_{i=1}^{n}f_{i}\cdot g_{y,i},\sum_{i=1}^{n}f_{i}\cdot gyx_{i},\sum_{i=1}^{n}f_{i}\cdot gyy_{i},\sum_{i=1}^{n}f_{i}\cdot gyz_{i}, \end{array}\\ \begin{array}{c} \sum_{i=1}^{n}f_{i}\cdot g_{z,i},\sum_{i=1}^{n}f_{i}\cdot gzx_{i},\sum_{i=1}^{n}f_{i}\cdot gzy_{i},\sum_{i=1}^{n}f_{i}\cdot gzz_{i},\sum_{i=1}^{n}f_{i},\sum_{i=1}^{n}f_{i}\cdot g_{i} \end{array} \end{array}$$

To evaluate the computational effort, the number of additions and multiplications are counted:

- Multiplications per voxel:
  − Nine multiplications in terms of Equation (29).
  − 86 multiplications in the 100 terms (Equation (32), inside the sums).
- Additions per voxel:
  − 100 additions in the 100 sums (Equation (32)).
- Further multiplications:
  − Computation of the product $r_{1}\cdot r_{1}$.
  − 84 multiplications of the prefactors with the sums in Equation (26) for the normal matrix.
  − 40 multiplications for the right hand side (Equation (31)) outside of the sums.
- Further additions:
  − 28 additions for the right hand side (Equation (31)).

Altogether, for the direct computation of $A^{T}\cdot A$ and $A^{T}\cdot l$, 95 multiplications and 100 additions per voxel are required. The additional multiplication and summations for building up $A^{T}\cdot A$ and $A^{T}\cdot l$ can be neglected due to the large number of voxel.

#### 2.4.2. Computational Costs of the Standard Method

The computational effort of the standard method contains building up the Jacobian matrix A and the reduced observation vector l as well as the matrix-matrix multiplication and the matrix-vector multiplication.

The following terms are calculated and saved in temporal variables for each line of the matrix A (for each voxel):

(33) $$\begin{array}{c} r1gx_{i}=r_{1}\cdot g_{x,i}\\ r1gy_{i}=r_{1}\cdot g_{y,i}\\ r1gz_{i}=r_{1}\cdot g_{z,i} \end{array}$$

For the each line of matrix A, the following nine terms have to be computed:

(34) $$\begin{array}{c} r1gx_{i}\cdot {\tilde{x}}_{i};r1gx_{i}\cdot {\tilde{y}}_{i};r1gx_{i}\cdot {\tilde{z}}_{i};r1gy_{i}\cdot {\tilde{x}}_{i};r1gy_{i}\cdot {\tilde{y}}_{i};r1gy_{i}\cdot {\tilde{z}}_{i};r1gz_{i}\cdot {\tilde{x}}_{i};r1gz_{i}\cdot {\tilde{y}}_{i};r1gz_{i}\cdot {\tilde{z}}_{i} \end{array}$$

For each element of l, there are one multiplication and two additions (subtractions):

(35) $$f_{i}-r_{0}-r_{1}\cdot g_{i}$$

- Multiplications per voxel:
  − One multiplication for building up the reduced observation vector l.
  − Three multiplications for the terms of Equation (33) that are computed temporally due to multiple use for building up the Jacobian matrix.
  − Nine further multiplications for building up the Jacobian matrix of Equation (34).
  − 105 multiplications (105 elements) due to the matrix multiplication of $A^{T}\cdot A$ (only upper triangular matrix).
  − 14 multiplications (14 elements) due to the matrix-vector multiplication of $A^{T}\cdot l$.

- Additions per voxel:
  − Two additions for building up the reduced observation vector l.
  − 105 additions (105 elements) due to the matrix multiplication of $A^{T}\cdot A$ (only upper triangular matrix).
  − 14 additions (14 elements) due to the matrix-vector multiplication of $A^{T}\cdot l$.

Altogether, for building up A and l and for the matrix multiplications $A^{T}\cdot A$ and $A^{T}\cdot l$, 132 multiplications and 121 additions per voxel are required.

#### 2.4.3. Theoretical Comparison

Table 1 summarizes the consideration and shows the number of multiplications and additions per voxel for the standard method and the direct computation of the normal equations. The direct method reduces the number of multiplications by 28% and reduces the number of additions by 17%. Appendix B and Appendix C also consider the cases that the radiometric parameters are fixed and that these parameters are omitted. In addition to the reduced number of multiplications and summations, the direct method also save memory because the computation of the n×14 Jacobian matrix (A) and n×1 observation vector (l) is avoided.

#### 2.4.4. Comparison of Time Measurements in Practise

The comparison was done on a desktop machine with a AMD Ryzen 7 3800X 8-Cores (16 threads) processor at 3.9 GHz. In the test, 3D LSM was applied to 146,933 cuboids defined in the first two data sets of an in-situ tension test of Lorenzoni et al. [18]. The patch dimension was set to 15×15×15 vx. The algorithm is implemented in C++ (using the GNU Compiler Collection, https://gcc.gnu.org) and compiled with the optimization option -O3. The loop over the matching points with the computation of the 3D LSM is parallelized with the OpenMP library (16 parallel units), and the time measurement only considers this loop. The time measurement includes the whole 3D LSM process so that not only the calculation of the normal matrix $A^{T}\cdot A$ and the right hand side vector $A^{T}\cdot l$ is analyzed. However, the computation of the normal equations is one of the most time-consuming steps herein.

Different calculation modes are tested: Method M1 stands for the direct computation. M2 represents the standard computation with building up the Jacobian matrix A and the reduced observation vector l as well as the matrix multiplications (only upper triangular matrix of the normal matrix) with an own implementation. M3 and M4 are similar to M2, but the matrix multiplications are done using the Eigen library. Eigen is a C++ template library for linear algebra (release 3.4.0, https://eigen.tuxfamily.org/). For M3, the full matrix of $A^{T}\cdot A$ is computed, and for M4, only the upper triangular matrix is computed. For all modes, before the calculation of the normal matrix begins, the values of the interpolated gray values and derivatives are computed and saved in separate arrays. All modes lead to the same numerical results, only the calculation times differ. Table 2 shows the calculation times for the different modes. The direct method is the fastest variant and almost twice as fast as the own standard computation M2. Compared to the best matrix multiplication variant M4 using Eigen (only upper triangular matrix), the direct method reduces the computation time by 27%. This reduction approximately agrees with the theoretical decrease of the multiplications of 28% (Section 2.4.3). The computation time difference between M3 and M4 is very small (only 9% decrease), although significantly more matrix elements (91) have to be computed (M3: 132 multiplications and 121 addition per voxel, M4: 223 multiplications and 212 addition per voxel).

## 3. Conclusions

The paper presents an optimized algorithm to compute 3D least-squares matching in voxel data sequences including geometric and radiometric parameters. Compared to the standard method of the estimation of the normal equations, the number of multiplications is reduced by 28% and the number of additions is reduced by 17%. In a practical test, the computation time was decreased by 27%.

## Figures and Tables

**Table 1:** Comparison of the computational effort between the direct calculation of the normal equations and the standard method.

Method	Multiplications per vx	Additions per vx
Standard	132	121
Direct	95	100

**Table 2:** Comparison of the computation times with different modes for the calculation of the normal equations.

Mode 1	M1	M2	M3	M4
Computation time [s]	12.8	23.2	19.3	17.5

## Data Availability

Not applicable.

## Abbreviations

The following abbreviations are used in this manuscript:

LSM	least-squares matching
vx	voxel
DVC	digital volume correlation
SHCC	strain-hardening cement-based composites
FFT	Fast Fourier Transform

## Appendix A. Optimized 3D Cross-Correlation

In the following, an optimized method of the 3D cross-correlation is presented. In our approach, the normalized cross-correlation coefficient is used, see Equation (A2). *t* is the template, the subvolume of the reference volume, to be matched. The number of voxel of the template is

(A1) $$n_{cc}=dim_{x}\cdot dim_{y}\cdot dim_{z}$$

(A2) $$\gamma_{x+h_{x},y+h_{y},z+h_{z}}=\frac{\sum_{i,j,k}{\hat{g}}_{x,y,z,i,j,k}\cdot {\hat{t}}_{i,j,k}}{\sqrt{\sum_{i,j,k}{\hat{g}}_{x,y,z,i,j,k}^{2}}\cdot \sqrt{\sum_{i,j,k}{\hat{t}}_{i,j,k}^{2}}}$$

where

${\hat{g}}_{x,y,z,i,j,k}=g_{x+i-1,y+j-1,z+k-1}-{\bar{g}}_{x+h_{x},y+h_{y},z+h_{z}}$
${\bar{g}}_{x+h_{x},y+h_{y},z+h_{z}}=\frac{1}{n_{cc}}\cdot \sum_{i,j,k}g_{x{+}_{i}-1,y+j-1,z+k-1}$
${\hat{t}}_{i,j,k}=t_{i,j,k}-\bar{t}$
$\bar{t}=\frac{1}{n_{cc}}\cdot \sum_{i,j,k}t_{i,j,k};\;\mathrm{mean}\mathrm{intensity}(template)$
$\sum_{i,j,k}\to \sum_{dim_{x}}^{i{=}^{1}}\;\sum_{dim_{y}}^{j{=}^{1}}\;\sum_{dim_{z}}^{k{=}^{1}}$

The terms of Equation (A2) are transformed as follows:

(A3) $$\begin{array}{c} \begin{array}{c} \sum_{i,j,k}{\hat{g}}_{x,y,z,i,j,k}\cdot {\hat{t}}_{i,j,k}=\sum_{i,j,k}\left[g_{x+i-1,y+j-1,z+k-1}\cdot t_{i,j,k}-g_{x+i-1,y+j-1,z+k-1}\cdot \bar{t}-t_{i,j,k}\cdot {\bar{g}}_{x+h_{x},y+h_{y},z+h_{z}}+\bar{t}\cdot {\bar{g}}_{x+h_{x},y+h_{y},z+h_{z}}\right]\\ =\sum_{i,j,k}g_{x+i-1,y+j-1,z+k-1}\cdot t_{i,j,k}-n_{cc}\cdot \bar{t}\cdot {\bar{g}}_{x+h_{x},y+h_{y},z+h_{z}} \end{array} \end{array}$$

(A4) $$\sum_{i,j,k}{\hat{g}}_{x,y,z,i,j,k}^{2}=\sum_{i,j,k}g_{x+i-1,y+j-1,z+k-1}^{2}-n_{cc}\cdot {\bar{g}}_{x+h_{x},y+h_{y},z+h_{z}}^{2}$$

(A5) $$\sum_{i,j,k}{\hat{t}}_{i,j,k}^{2}=\sum_{i,j,k}t_{i,j,k}^{2}-2\cdot \sum_{i,j,k}t_{i,j,k}\cdot \bar{t}+\sum_{i,j,k}{\bar{t}}^{2}=\sum_{i,j,k}t_{i,j,k}^{2}-n_{cc}\cdot {\bar{t}}^{2}$$

There are optimized methods to compute the transformed terms using two approaches:

- The values of $\sum_{i,j,k}g_{x+i-1,y+j-1,z+k-1}\cdot t_{i,j,k}$ are computed for the whole search area using the 3D Fast Fourier transform as also done by Lewis [19] for 2D and Yang et al. [14] for 3D: $F^{-1}\left\{F\left(g\right)\circ F^{*}\left(t^{\prime }\right)\right\}$ where $F^{*}$ are the complex conjugate values (∘: Hadamard product, element-wise multiplication). $t^{\prime }$ is a volume data set with the same size as the search volume containing *t* and is filled up with zeros for indices exceeding the dimensions of *t*.
- The values of ${\bar{g}}_{x+h_{x},y+h_{y},z+h_{z}}$ and $\sum_{i,j,k}g_{x+i-1,y+j-1,z+k-1}^{2}$ are computed using the integral volume method [20].

The terms $\sum_{i,j,k}t_{i,j,k}^{2}$ and $\bar{t}$ are computed straight forward. The position of the maximum coefficient $\underset{x,y,z}{max}\gamma_{x+h_{x},y+h_{y},z+h_{z}}$ can be used as initial position for the 3D LSM.

## Appendix B. 3D Least-Squares Matching with Fixed Radiometric Parameters

This section considers the case that the radiometric parameters are fixed and shows the most important changes.

### Appendix B.1. Mathematical Model

The relationship between the gray values of the reference and the second volume stays the same as before, see Equation (2).

(A6) $$f\left(x_{Ref},y_{Ref},z_{Ref}\right)=r_{0}+r_{1}\cdot g\left(x_{Def},y_{Def},z_{Def}\right)$$

The vector of unknowns reduces to the affine parameters:

(A7) $$p={\left(\begin{array}{cccccccccccc} a_{0}, & a_{1}, & a_{2}, & a_{3}, & b_{0}, & b_{1}, & b_{2}, & b_{3}, & c_{0}, & c_{1}, & c_{2}, & c_{3} \end{array}\right)}^{T}$$

In the case of fixed radiometric parameters, the last two columns of the Jacobian matrix A are omitted compared to Equation (14) (for $A_{1},A_{2}$ and $A_{3}$ see Equations (15) to (17)):

(A8) $$A=\left(\begin{array}{ccc} A_{1} & A_{2} & A_{3} \end{array}\right)$$

The reduced observation vector l does not change:

(A9) $$l=\left(\begin{array}{c} f_{1}-r_{0}-r_{1}\cdot g_{1}\\ f_{2}-r_{0}-r_{1}\cdot g_{2}\\ \vdots \\ f_{n}-r_{0}-r_{1}\cdot g_{n} \end{array}\right)$$

Thus, the normal matrix $A^{T}\cdot A$ and the right hand side $A^{T}\cdot l$ are also reduced compared to Equations (26) to (31):

(A10) $$A^{T}\cdot A=\left(\begin{array}{ccc} \begin{array}{c} A_{1}^{T}\cdot A_{1} \end{array} & A_{1}^{T}\cdot A_{2} & A_{1}^{T}\cdot A_{3}\\ \; & A_{2}^{T}\cdot A_{2} & A_{2}^{T}\cdot A_{3}\\ \; & \; & A_{3}^{T}\cdot A_{3} \end{array}\right)=\left(\begin{array}{ccc} r_{1}^{2}\cdot \sum_{i=1}^{n}G_{11,i} & r_{1}^{2}\cdot \sum_{i=1}^{n}G_{12,i}\; & r_{1}^{2}\cdot \sum_{i=1}^{n}G_{13,i}\\ \; & r_{1}^{2}\cdot \sum_{i=1}^{n}G_{22,i} & r_{1}^{2}\cdot \sum_{i=1}^{n}G_{23,i}\\ \; & \; & r_{1}^{2}\cdot \sum_{i=1}^{n}G_{33,i} \end{array}\right)$$

(A11) $$A^{T}\cdot l=\left(\begin{array}{c} \begin{array}{c} A_{1}^{T}\cdot l \end{array}\\ A_{2}^{T}\cdot l\\ A_{3}^{T}\cdot l \end{array}\right)=\left(\begin{array}{c} r_{1}\cdot \sum_{i=1}^{n}df_{i}\cdot g_{x,i}\\ r_{1}\cdot \sum_{i=1}^{n}df_{i}\cdot gxx_{i}\\ r_{1}\cdot \sum_{i=1}^{n}df_{i}\cdot gxy_{i}\\ r_{1}\cdot \sum_{i=1}^{n}df_{i}\cdot gxz_{i}\\ r_{1}\cdot \sum_{i=1}^{n}df_{i}\cdot g_{y,i}\\ r_{1}\cdot \sum_{i=1}^{n}df_{i}\cdot gyx_{i}\\ r_{1}\cdot \sum_{i=1}^{n}df_{i}\cdot gyy_{i}\\ r_{1}\cdot \sum_{i=1}^{n}df_{i}\cdot gyz_{i}\\ r_{1}\cdot \sum_{i=1}^{n}df_{i}\cdot g_{z,i}\\ r_{1}\cdot \sum_{i=1}^{n}df_{i}\cdot gzx_{i}\\ r_{1}\cdot \sum_{i=1}^{n}df_{i}\cdot gzy_{i}\\ r_{1}\cdot \sum_{i=1}^{n}df_{i}\cdot gzz_{i} \end{array}\right)$$

where $df_{i}=f_{i}-r_{0}-r_{1}\cdot g_{i}$

### Appendix B.2. Computational Effort

#### Appendix B.2.1. Direct Method

As above, for each voxel, the following terms are computed and saved in temporal variables because they are used several times:

(A12) $$\begin{array}{c} gxx_{i}=g_{x,i}\cdot {\tilde{x}}_{i}\\ gxy_{i}=g_{x,i}\cdot {\tilde{y}}_{i}\\ gxz_{i}=g_{x,i}\cdot {\tilde{z}}_{i}\\ gyx_{i}=g_{y,i}\cdot {\tilde{x}}_{i}\\ gyy_{i}=g_{y,i}\cdot {\tilde{y}}_{i}\\ gyz_{i}=g_{y,i}\cdot {\tilde{z}}_{i}\\ gzx_{i}=g_{z,i}\cdot {\tilde{x}}_{i}\\ gzy_{i}=g_{z,i}\cdot {\tilde{y}}_{i}\\ gzz_{i}=g_{z,i}\cdot {\tilde{z}}_{i}\\ df_{i}=f_{i}-r_{0}-r_{1}\cdot g_{i} \end{array}$$

60 terms of the matrices $G_{11}$, $G_{12}$, $G_{13}$, $G_{22}$, $G_{23}$ and $G_{33}$ (Equation (30)) and 12 terms of $A^{T}\cdot l$ are computed. To evaluate the computational effort of the direct method, the number of additions and multiplications are counted. Only the operations per voxel are considered, the other operations can be neglected.

- Multiplications per voxel:
  − Ten multiplications for the temporally terms of Equation (A12).
  − 60 multiplications in the $G_{ij}$ matrices.
  − 12 multiplications in the sums of $A^{T}\cdot l$.
- Additions per voxel:
  − Two additions (subtractions) in $df_{i}=f_{i}-r_{0}-r_{1}\cdot g_{i}$.
  − 60 additions in the 60 sums of the $G_{ij}$ matrices.
  − 12 additions in the 12 sums of $A^{T}\cdot l$.

All in all, 82 multiplications and 74 additions have to be computed per voxel for the direct method.

#### Appendix B.2.2. Standard Method

For the standard method, building up A and l as well as the matrix multiplications are considered:

- Multiplications per voxel:
  − One multiplication for building up the reduced observation vector l.
  − Three multiplications for the terms of Equation (33) that are computed temporally due to multiple use for building up the Jacobian matrix.
  − Nine further multiplications for building up the Jacobian matrix of Equation (34).
  − 78 multiplications (78 elements) due to the matrix multiplication of $A^{T}\cdot A$ (only upper triangular matrix).
  − 12 multiplications (12 elements) due to the matrix-vector multiplication of $A^{T}\cdot l$.

- Additions per voxel:
  − Two additions (subtractions) for building up the reduced observation vector l.
  − 78 additions (78 elements) due to the matrix multiplication of $A^{T}\cdot A$ (only upper triangular matrix).
  − 12 additions (12 elements) due to the matrix-vector multiplication of $A^{T}\cdot l$.

Thus, for the standard method, 103 multiplications and 92 additions have to be computed per voxel.

#### Appendix B.2.3. Comparison

Table A1 shows the number of computations per voxel for the direct and the standard method. Using the direct method, 20.4% less multiplications and 19.6% less additions are done.

**Table A1:** Comparison of the computational effort between the direct computation of the normal equations and the standard method (affine mathematical model with fixed radiometric parameters).

Method	Multiplications per vx	Additions per vx
Standard	103	92
Direct	82	74

## Appendix C. 3D Least-Squares Matching with 12-Parameter Affine Transformation

In this section, only the affine transformation with 12 parameters is used as mathematical model.

### Appendix C.1. Mathematical Model

The relationship between the gray values of the reference and the second volume is reduced to:

(A13) $$f\left(x_{Ref},y_{Ref},z_{Ref}\right)=g\left(x_{Def},y_{Def},z_{Def}\right)$$

The vector of unknowns is the same as in the case of Appendix B:

(A14) $$p={\left(\begin{array}{cccccccccccc} a_{0}, & a_{1}, & a_{2}, & a_{3}, & b_{0}, & b_{1}, & b_{2}, & b_{3}, & c_{0}, & c_{1}, & c_{2}, & c_{3} \end{array}\right)}^{T}$$

In the Jacobian matrix A, the $r_{1}$ parameter is omitted and also the reduced observation vector l differs:

(A15) $$A=\left(\begin{array}{ccc} A_{1} & A_{2} & A_{3} \end{array}\right)$$

with the submatrices:

(A16) $$A_{1}=\left(\begin{array}{cccc} \begin{array}{c} \frac{\partial f}{\partial a_{0}}{|}_{p=p_{0}} \end{array} & \frac{\partial f}{\partial a_{1}}{|}_{p=p_{0}} & \frac{\partial f}{\partial a_{2}}{|}_{p=p_{0}} & \frac{\partial f}{\partial a_{3}}{|}_{p=p_{0}}\\ \vdots & \vdots & \vdots & \vdots \end{array}\right)=\left(\begin{array}{cccc} g_{x,1} & g_{x,1}\cdot {\tilde{x}}_{1} & g_{x,1}\cdot {\tilde{y}}_{1} & g_{x,1}\cdot {\tilde{z}}_{1}\\ g_{x,2} & g_{x,2}\cdot {\tilde{x}}_{2} & g_{x,2}\cdot {\tilde{y}}_{2} & g_{x,2}\cdot {\tilde{z}}_{2}\\ \vdots & \vdots & \vdots & \vdots \\ g_{x,n} & g_{x,n}\cdot {\tilde{x}}_{n} & g_{x,n}\cdot {\tilde{y}}_{n} & g_{x,n}\cdot {\tilde{z}}_{n} \end{array}\right)$$

(A17) $$A_{2}=\left(\begin{array}{cccc} \begin{array}{c} \frac{\partial f}{\partial b_{0}}{|}_{p=p_{0}} \end{array} & \frac{\partial f}{\partial b_{1}}{|}_{p=p_{0}} & \frac{\partial f}{\partial b_{2}}{|}_{p=p_{0}} & \frac{\partial f}{\partial b_{3}}{|}_{p=p_{0}}\\ \vdots & \vdots & \vdots & \vdots \\ g_{x,n} & g_{x,n}\cdot {\tilde{x}}_{n} & g_{x,n}\cdot {\tilde{y}}_{n} & g_{x,n}\cdot {\tilde{z}}_{n} \end{array}\right)=\left(\begin{array}{cccc} g_{y,1} & g_{y,1}\cdot {\tilde{x}}_{1} & g_{y,1}\cdot {\tilde{y}}_{1} & g_{y,1}\cdot {\tilde{z}}_{1}\\ g_{y,2} & g_{y,2}\cdot {\tilde{x}}_{2} & g_{y,2}\cdot {\tilde{y}}_{2} & g_{y,2}\cdot {\tilde{z}}_{2}\\ \vdots & \vdots & \vdots & \vdots \\ g_{y,n} & g_{y,n}\cdot {\tilde{x}}_{n} & g_{y,n}\cdot {\tilde{y}}_{n} & g_{y,n}\cdot {\tilde{z}}_{n} \end{array}\right)$$

(A18) $$A_{3}=\left(\begin{array}{cccc} \begin{array}{c} \frac{\partial f}{\partial c_{0}}{|}_{p=p_{0}} \end{array} & \frac{\partial f}{\partial c_{1}}{|}_{p=p_{0}} & \frac{\partial f}{\partial c_{2}}{|}_{p=p_{0}} & \frac{\partial f}{\partial c_{3}}{|}_{p=p_{0}}\\ \vdots & \vdots & \vdots & \vdots \end{array}\right)=\left(\begin{array}{cccc} g_{z,1} & g_{z,1}\cdot {\tilde{x}}_{1} & g_{z,1}\cdot {\tilde{y}}_{1} & g_{z,1}\cdot {\tilde{z}}_{1}\\ g_{z,2} & g_{z,2}\cdot {\tilde{x}}_{2} & g_{z,2}\cdot {\tilde{y}}_{2} & g_{z,2}\cdot {\tilde{z}}_{2}\\ \vdots & \vdots & \vdots & \vdots \\ g_{z,n} & g_{z,n}\cdot {\tilde{x}}_{n} & g_{z,n}\cdot {\tilde{y}}_{n} & g_{z,n}\cdot {\tilde{z}}_{n} \end{array}\right)$$

(A19) $$l=\left(\begin{array}{c} f_{1}-g_{1}\\ f_{2}-g_{2}\\ \vdots \\ f_{n}-g_{n} \end{array}\right)$$

The normal matrix $A^{T}\cdot A$ (with the $G_{kl,i}$ matrices from Equations (27) and (30)) is:

(A20) $$A^{T}\cdot A=\left(\begin{array}{ccc} \begin{array}{c} A_{1}^{T}\cdot A_{1} \end{array} & A_{1}^{T}\cdot A_{2} & A_{1}^{T}\cdot A_{3}\\ \; & A_{2}^{T}\cdot A_{2} & A_{2}^{T}\cdot A_{3}\\ \; & \; & A_{3}^{T}\cdot A_{3} \end{array}\right)=\left(\begin{array}{ccc} \sum_{i=1}^{n}G_{11,i} & \sum_{i=1}^{n}G_{12,i}\; & \sum_{i=1}^{n}G_{13,i}\\ \; & \sum_{i=1}^{n}G_{22,i}\; & \sum_{i=1}^{n}G_{23,i}\\ \; & \; & \sum_{i=1}^{n}G_{33,i} \end{array}\right)$$

The right hand side of the normal equations simplifies to:

(A21) $$A^{T}\cdot l=\left(\begin{array}{c} \begin{array}{c} A_{1}^{T}\cdot l \end{array}\\ A_{2}^{T}\cdot l\\ A_{3}^{T}\cdot l \end{array}\right)=\left(\begin{array}{c} \sum_{i=1}^{n}df_{i}\cdot g_{x,i}\\ \sum_{i=1}^{n}df_{i}\cdot gxx_{i}\\ \sum_{i=1}^{n}df_{i}\cdot gxy_{i}\\ \sum_{i=1}^{n}df_{i}\cdot gxz_{i}\\ \sum_{i=1}^{n}df_{i}\cdot g_{y,i}\\ \sum_{i=1}^{n}df_{i}\cdot gyx_{i}\\ \sum_{i=1}^{n}df_{i}\cdot gyy_{i}\\ \sum_{i=1}^{n}df_{i}\cdot gyz_{i}\\ \sum_{i=1}^{n}df_{i}\cdot g_{z,i}\\ \sum_{i=1}^{n}df_{i}\cdot gzx_{i}\\ \sum_{i=1}^{n}df_{i}\cdot gzy_{i}\\ \sum_{i=1}^{n}df_{i}\cdot gzz_{i} \end{array}\right)$$

where $df_{i}=f_{i}-g_{i}$

### Appendix C.2. Computational Effort

#### Appendix C.2.1. Direct Method

Similar to the case above, for each voxel, the following terms are computed and saved in temporal variables because they are used several times:

(A22) $$\begin{array}{c} gxx_{i}=g_{x,i}\cdot {\tilde{x}}_{i}\\ gxy_{i}=g_{x,i}\cdot {\tilde{y}}_{i}\\ gxz_{i}=g_{x,i}\cdot {\tilde{z}}_{i}\\ gyx_{i}=g_{y,i}\cdot {\tilde{x}}_{i}\\ gyy_{i}=g_{y,i}\cdot {\tilde{y}}_{i}\\ gyz_{i}=g_{y,i}\cdot {\tilde{z}}_{i}\\ gzx_{i}=g_{z,i}\cdot {\tilde{x}}_{i}\\ gzy_{i}=g_{z,i}\cdot {\tilde{y}}_{i}\\ gzz_{i}=g_{z,i}\cdot {\tilde{z}}_{i}\\ df_{i}=f_{i}-g_{i} \end{array}$$

60 terms of the matrices $G_{11}$, $G_{12}$, $G_{13}$, $G_{22}$, $G_{23}$ and $G_{33}$ (Equation (30)) and 12 terms of $A^{T}\cdot l$ are computed. To evaluate the computational effort of the direct method, the number of additions and multiplications are counted. Only the operations per voxel are considered, the other operations can be neglected.

- Multiplications per voxel:
  − Nine multiplications for the temporally terms of Equation (A22).
  − 60 multiplications in the $G_{ij}$ matrices.
  − 12 multiplications in the sums of $A^{T}\cdot l$.
- Additions per voxel:
  − One addition (subtraction) in $df_{i}=f_{i}-g_{i}$.
  − 60 additions in the 60 sums of the $G_{ij}$ matrices.
  − 12 additions in the 12 sums of $A^{T}\cdot l$.

All in all, 81 multiplications and 73 additions have to be computed per voxel for the direct method.

#### Appendix C.2.2. Standard Method

For the standard method, the buildup of A and l as well as the matrix multiplications are considered:

- Multiplications per voxel:
  − Nine multiplications for building up the Jacobian matrix of Equation (A15).
  − 78 multiplications (78 elements) due to the matrix multiplication of $A^{T}\cdot A$ (only upper triangular matrix).
  − 12 multiplications (12 elements) due to the matrix-vector multiplication of $A^{T}\cdot l$.

- Additions per voxel:
  − One addition (subtraction) for building up the reduced observation vector l.
  − 78 additions (78 elements) due to the matrix multiplication of $A^{T}\cdot A$ (only upper triangular matrix).
  − 12 additions (12 elements) due to the matrix-vector multiplication of $A^{T}\cdot l$.

Thus, for the standard method, 99 multiplications and 91 additions have to be computed per voxel.

#### Appendix C.2.3. Comparison

Table A2 shows the number of computations per voxel for the direct and the standard method. Using the direct method, 18.2% less multiplications and 19.8% less additions are done.

**Table A2:** Comparison of the computational effort between the direct computation of the normal equations and the standard method (affine mathematical model without radiometric parameters).

Method	Multiplications per vx	Additions per vx
Standard	99	91
Direct	81	73
